# Harnessing the Power of Induced Pluripotent Stem Cells and Gene Editing Technology: Therapeutic Implications in Hematological Malignancies

**DOI:** 10.3390/cells10102698

**Published:** 2021-10-09

**Authors:** Ishnoor Sidhu, Sonali P. Barwe, Raju K. Pillai, Anilkumar Gopalakrishnapillai

**Affiliations:** 1 Nemours Centers for Childhood Cancer Research and Cancer & Blood Disorders, Nemours Children’s Health, Wilmington, DE 19803, USA; ishnoor@udel.edu (I.S.); sbarwe@nemours.org (S.P.B.); 2 Department of Biological Sciences, University of Delaware, Newark, DE 19711, USA; 3 National Medical Center, Department of Pathology, City of Hope, Duarte, CA 91105, USA; rpillai@coh.org

**Keywords:** iPSC, gene editing, hematologic

## Abstract

In vitro modeling of hematological malignancies not only provides insights into the influence of genetic aberrations on cellular and molecular mechanisms involved in disease progression but also aids development and evaluation of therapeutic agents. Owing to their self-renewal and differentiation capacity, induced pluripotent stem cells (iPSCs) have emerged as a potential source of short in supply disease-specific human cells of the hematopoietic lineage. Patient-derived iPSCs can recapitulate the disease severity and spectrum of prognosis dictated by the genetic variation among patients and can be used for drug screening and studying clonal evolution. However, this approach lacks the ability to model the early phases of the disease leading to cancer. The advent of genetic editing technology has promoted the generation of precise isogenic iPSC disease models to address questions regarding the underlying genetic mechanism of disease initiation and progression. In this review, we discuss the use of iPSC disease modeling in hematological diseases, where there is lack of patient sample availability and/or difficulty of engraftment to generate animal models. Furthermore, we describe the power of combining iPSC and precise gene editing to elucidate the underlying mechanism of initiation and progression of various hematological malignancies. Finally, we discuss the power of iPSC disease modeling in developing and testing novel therapies in a high throughput setting.

## 1. Introduction

Disease models provide a useful tool to deconstruct the alterations in the biological processes that lead to various kinds of hematological malignancies such as leukemia, lymphoma and multiple myeloma. To date, murine models constitute the mainframe for studying hematological malignancies and testing therapeutic agents (reviewed in detail recently [1,2,3]). The relevance of carcinogen-induced [4] or virus-induced animal models [5] in human malignancies is limited. Different types of mouse models such as knock-in models, knock-out models, and conditional knock-in/knock-out models (by using Tet on/off system or CRE/Lox system), enable the study of gene-specific effects in a tissue- or developmental stage-specific manner. Patient-derived xenograft models are beneficial in studying human disease in the mouse system and are used for preclinical drug evaluation [6]. Some of the recently generated animal models for leukemia, lymphoma and multiple myeloma are listed in Table 1.

Although murine models have substantially contributed to our understanding of hematological malignancies, there are some limitations associated with these models. Firstly, the failure of recapitulation of disease features in mice due to absence of all human gene orthologs. For example, the partial trisomy 21 murine models failed to develop Down syndrome-myeloid leukemia (DS-ML) likely due to lack of missing orthologs of Hsa21 in mice, specifically the micro-RNAs [25,26]. Since mice do not have many of the trisomy 21 genes, the forceful expression of these HSa21 genes may not show a similar phenotype in mice and therefore will not be the right model to understand the initiation and progression of DS-ML. Secondly, many therapeutic drugs that have shown excellent efficacy in murine or other animal models have failed in human trials. For example, the CD28 superagonist antibody TGN1412 which passed the pre-clinical testing in non-human primates, did not show appreciable efficacy in phase I clinical trial [27]. Likewise, many drugs have failed in real-life clinical trials [28,29]. In addition, understanding the disease progression in murine models requires euthanizing and analyzing multiple mice at various time points. This presents with its own disadvantages; namely increase in number of animals which in turn increase the cost and the variability.

To circumvent some of these disadvantages, researchers have invested their time in developing zebrafish models for hematology and related disorders, which are reviewed recently [30,31]. Besides the advantages of easy maintenance, high fecundity, high conservation of key developmental processes, the opacity of zebrafish provides the ability to visualize and trace the fate of malignant cells. Transgenic zebrafish models have been used to understand the genetic lesions responsible for hematological malignancies [32,33,34,35,36]. Additionally, the zebrafish xenograft model has been shown to provide a cost-effective alternative to drug optimization and development of personalized medicine for leukemia [37,38] and multiple myeloma [39]. Despite these advantages, zebrafish are anatomically very distant from humans.

Due to the failure to recapitulate all of the disease features owing to lack of human orthologs in mice and zebrafish, human cell models are gaining momentum. Blood cancer-derived immortalized cell lines are used as an alternative to murine models [40,41]. However, the method of immortalization may alter the properties of the primary patient sample in immortalized cell lines adapted for *in vitro* growth. Primary patient samples and patient-derived xenograft mice are good for studying hematological malignancies, but the paucity of samples is rate limiting in the development of wide spectrum disease models. Functional studies to understand the gene-specific effects are restricted by the relative inability of primary patient cells to grow *in vitro* for longer periods of time. In addition, patient samples cannot be used for initiation and progression of the disease since the samples are collected at the time of diagnosis, relapse or a terminal stage.

The advent of human embryonic stem cells in 1998 [42] has been the basis of reprogramming fibroblasts into iPSCs [43]. By the overexpression of transcription factors (such as OCT4, SOX2, NANOG, LIN28, KLF4, MYC), human fetal, neonatal and adult primary cells were reprogrammed to generate iPSCs [44,45,46]. The use of non-integrational methods such as episomal vectors, Sendai virus or mRNA have alleviated the risk of insertional mutagenesis while achieving high reprograming efficiencies [47,48,49]. The use of reprogrammed induced pluripotent stem cells (iPSCs) has shown promise for the generation of custom-tailored cells for modeling hematological malignancies and drug screening [50]. Their self-renewable property and the potential for differentiation into hematopoietic stem and progenitor cells (HSPCs) make iPSCs a cost-effective and scalable approach for disease modeling. Direct conversion approaches to generate cells of the hematopoietic lineage from fibroblasts using a one-step approach or directed differentiation from human embryonic stem cells have been used as an alternative to somatic cell reprogramming followed by hematopoietic differentiation of iPSCs [51]. The use of iPSC model for hematological disorders with a focus on patient-specific iPSCs has been reviewed [50,52,53,54]. The generation of isogenic pairs of normal and mutated iPSC lines using gene editing methodology helps in understanding the key role of specific mutations. The current review focuses on the development of iPSC models for understanding initiation and progression of hematological malignancies with a primary focus on using genetically engineered iPSCs as an alternative to patient-derived iPSCs. While the importance of hematopoietic differentiation of iPSC in disease modeling has been reviewed elsewhere [53,54], the present review highlights the development of 3-dimensional culture protocols for *in vitro* differentiation of iPSCs towards hematopoietic lineage.

## 2. Disease Modeling Using Patient-Derived iPSCs

The understanding of human genetic disorders has been revolutionized with the development of patient-derived iPSCs especially for hematological diseases and cancer. The generation of iPSCs from human melanoma cells was first reported in 2008 [55]. Since then, various groups have established patient-derived iPSCs from hematological malignancies including acute myeloid leukemia (AML) [56], pediatric acute leukemias [57], myelodysplastic syndrome (MDS) [58], transient myeloproliferative disorder (TMD) [59,60], myeloproliferative neoplasm (MPN) [61], chronic myeloid leukemia (CML) [62,63], acute lymphoblastic leukemia (ALL) [64] and multiple myeloma (MM) [65].

It is well established that genetic mutations lie at the center of various hematological malignancies. The somatic and germ-line mutations in *RUNX1* in various hematological malignancies including pre-leukemic MDS have been observed in patient samples. However, the causative role of these mutations in AML predisposition cannot be studied using patient cells. Familial platelet disorder/AML (FPD/AML) is one such rare autosomal disease caused by a germline mutation in the *RUNX1* gene. The FPD/AML patients do not show clinical symptoms until they develop MDS or AML in the third decade of life. The FPD/AML patient-derived iPSC model has provided insight on the role of *RUNX1* mutation in the emergence of early HSPCs with a defect in megakaryocyte maturation. The study using FPD/AML-iPSCs further revealed the differential effect of heterozygous *RUNX1* mutation in mice vs. human, where no thrombocytopenia was observed in mice whereas it caused defects in *in vitro* generation of megakaryocytes in human iPSCs. Interestingly, in contrast to previously established dominant-negative effect, this study proposed a loss-of-function effect of the *RUNX1* mutation [66]. To resolve this contradiction, iPSCs generated from patient lines representing the dominant-negative mutation or monoallelic gene deletion were used to understand the role of *RUNX1* dosage on leukemia predisposition. Results from this study showed that *RUNX1* loss resulted in predisposition to leukemia whereas haploinsufficiency led to defects in primitive erythropoiesis and megakaryopoiesis, and caused thrombocytopenia with no leukemia [67].

Validation of the effect of driver mutations using patient-derived iPSCs is useful in the classification of disease subtypes and identification of targeted therapy. In order to study the dominant signaling pathway, patient-derived iPSCs have been very useful, especially in diseases that have fetal origin and affect young children such as juvenile myelomonocytic leukemia (JMML). Somatic or germline mutations in Ras pathway and associated genes (*PTPN11*, *CBL*, *NF1*, *KRAS* and *NRAS*) are implicated in JMML [68]. JMML patient-derived iPSCs harboring mutation in *PTPN11* showed increased bias towards myeloid differentiation and increased Granulocyte-Macrophage Colony-Stimulating Factor (GM-CSF) hypersensitivity [69]. Studying the effect of mutation in different category of genes in JMML, researchers developed iPSCs from JMML patient cells harboring either *PTPN11* mutation or *CBL* mutation. While the *PTPN11*-mutant showed a hyperactive Ras/MAPK pathway, *CBL*-mutant showed an aberrant activation of JAK/STAT pathway. The constitutive activation of PI3K/Akt/mTOR signaling pathway was observed in both mutants [70]. These studies aided in the development of targeted therapy options based on the genetic subtype of disease. The limitation of these studies was the lack of genetically similar and age matched control due to the paucity of the samples and the age of disease initiation.

Establishing isogenic iPSC lines from the patients containing the wild type and mutant clones helps understand the effect of mutations in the hematopoietic cell development in the genetic context. For example, isogenic iPSCs clones expressing JAK2-V617F mutant and wild type JAK2 were generated from polycythemia vera (PV) patients. The mutant iPSCs showed augmented erythropoiesis as compared to wild type cells *in vitro*. However, researchers were unable to determine whether the isogenic clone with wild type JAK2 was normal or a pre-mutant clone [71]. It is widely known that most of the hematological malignancies especially the leukemias have clonal heterogeneity due to acquisition of mutations in the pre-leukemic stage and progression to leukemic phase. While in patient cells capturing subclonal pre-leukemic population is difficult, reprogrammed iPSCs from single clones can generate a library of clones that can be utilized in studying the step-wise mutation acquisition and its effect on HSPCs self-renewal and differentiation [72,73].

From the decades of research, it has been established that most hematological malignancies have stepwise acquisition of genetic and cytogenetic abnormalities. The modeling of these sequential events can help unravel the role of each of these events in initiation and progression of the disease. Kotini et al. developed an induced pluripotent stem cell (iPSCs) panel derived from patients across the disease spectrum including familial predisposition, low-risk MDS, high-risk MDS and MDS/AML. The hematopoietic differentiation of this iPSC panel revealed the phenotypes of differentiation halt at different stages [74]. However, the diverse genetic backgrounds of patient-derived cell lines limited the power of genomic analysis. In the following sections, we will provide examples of how the customizable iPSCs have been utilized to generate a human de novo oncogenic model displaying each step during the disease initiation and progression.

The patient-derived iPSCs is advantageous in distinguishing the leukemia initiating cells from normal hematopoietic stem cells (HSCs) to identify novel diagnostic and targeted therapeutic markers. A recent study developed iPSC from leukemic stem cells (LSCs) and more matured blast cells and identified that iPSCs generated from LSCs showed higher engraftment potential and depended on RUNX1 for survival [75]. Further studies are warranted to identify markers for distinguishing LSCs from HSCs.

The rare hereditary disorder could benefit from iPSC generated from non-diseased somatic cells in studying the effect of the inherited mutations avoiding the genetic alterations acquired during disease progression. JMML in Noonan syndrome (NS/JMML) is a rare hereditary disorder caused by germline mutation in the *PTPN11* gene. The generation of iPSC from skin fibroblast and subsequent hematopoietic differentiation represented the early disease features such as hypersensitivity to GM-CSF and hyperproliferation of myeloid population. The study also identified micro-RNAs upregulated in NS/JMML as compared to NS (without JMML) and control making it a potential target for novel therapies [76].

Despite being valuable in understanding the mechanism of hematological pathogenesis, patient-derived iPSC models have certain limitations. The rarity of the samples that limits the use of patient cells directly, also affects the development of reprogrammed cells capturing the spectrum of disease specific mutations. Reprogramming patient cells often fails to capture earlier stages of disease. Moreover, the malignant genetic or cytogenetic transformations have shown to interfere in the reprogramming of disease cells. Attempts to reprogram cells from AML patients with high-risk translocations resulted in cytogenetically normal iPSCs [77,78] suggesting that not all genetic abnormalities can be preserved during reprogramming. This can be seen in relatively negligible reports of iPSC generations from fully malignant cells as compared to pre-malignant cells as in case of DS-ML and TMD. There have been few reports of generation of iPSCs from TMD [59,60] but no reports of iPSC generation from DS-ML cells. Lastly, patient-derived iPSCs often lack an appropriate isogenic control that could impede the discovery of novel therapeutic targets.

## 3. Disease Modeling Using Genetically Modified iPSCs

Studying the effect of a single oncogenic hit warrants the use of an initial cell type free of other mutations found in the patients. Reprogramming non-diseased cells and introducing disease-specific mutations by genome editing [79] is used to study these oncogenic events in isolation. Table 2 summarizes the genetically engineered iPSC models for studying the initiation and progression of hematological malignancies and identifying therapeutic targets for cure or blocking the progression to malignant stage. 

*RUNXI* mutation and chromosomal translocation resulting in the production of the RUNX1-RUNX1T1 fusion protein are found in two different types of AML with differing prognoses. The iPSCs harboring any of the two oncogenic (*RUNX1*-S291fs300X and induced expression of RUNX1-RUNX1T1) events had a block in granulocytic differentiation and enhanced self-renewal [81,82]. However, the transcriptomic profiling identified various differences in the target genes where the mutant RUNX1 can repress or induce target genes whereas RUNX1-RUNX1T1 fusion protein binding to targets led to repression of genes. The epigenomic profiling suggests more gene-specific regulation of transcription by mutant RUNX1 in contrast to more introns and intergenic binding by RUNX1-RUNX1T1 [81]. These studies have the advantage of non-interference of other mutations and showed the underlying contribution towards differences in clinical outcomes of the two types of leukemia.

The iPSCs harboring inducible mutations provide a tool to understand the effect of mutated genes devoid of other genetic alterations. Different mutations in the same signaling molecule can result in varying disease phenotype. The mechanism of two *JAK2* activating mutations in myeloproliferative neoplasms was deciphered using doxycycline-inducible customizable iPSC to ensure expression at the distinct time during the differentiation process. In contrast to *JAK2* V617F mutation that caused both erythrocytosis in PV and thrombocytosis in essential thrombocythemia (ET), *JAK2* exon12 N542-E543del (*JAK2*exon12) mutation caused only erythrocytosis in PV. The customized iPSCs showed the mutations activated different intracellular signals explaining the difference in the phenotype of patients [85].

Development of an iPSC-based initiation model, especially in diseases initiating *in utero*, can help explore the impact of oncogenes during development in a developmentally relevant human system. It is established that the childhood malignancies are clinically distinct from adult counterparts for example, the childhood affiliation of ETV6-RUNX1 fusion protein in childhood ALL (cALL). In an initiation model of cALL, hematopoietic differentiation of iPSCs expressing ETV6-RUNX1 produced proB cells with myeloid gene expression accompanied by a block in B lineage commitment which is specific during fetal development [86].

### 3.1. Clonal Evolution of AML: An Example of De Novo Leukemogenesis in Human iPSCs

**Figure 1:**
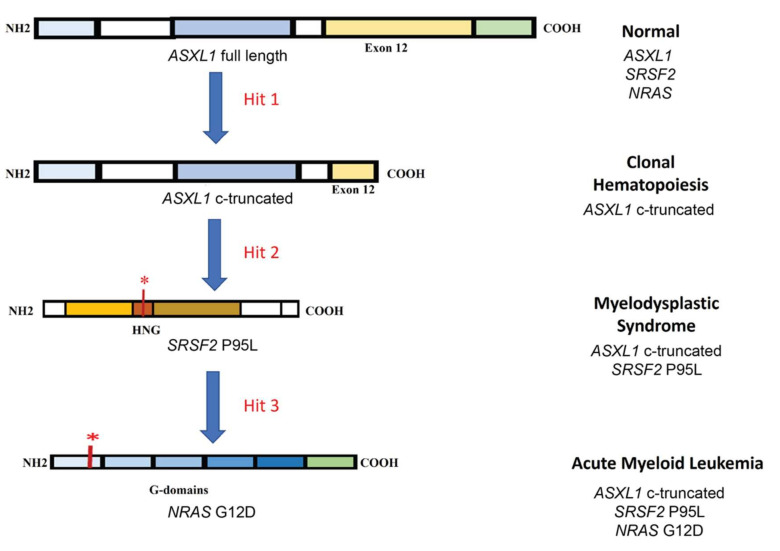
Schematic of clonal evolution of AML. The first step in the process is mutation in transcriptional regulator (*ASXL1*) resulting in a c-terminal truncated protein. The next step is a point mutation in hinge region (HGN) of the RNA splicing regulator (*SRSF2*) affecting the protein binding to canonical splicing enhancer sequences in RNA. The third hit is mutation in signaling molecule (*NRAS*) resulting in differentiation block and hyperproliferation. * indicates mutation.

The clonal evolution of AML is a multi-step transformation from normal to malignant stage via pre-malignant steps including clonal hematopoiesis (CH) and MDS. Unlike other cancers, AML has been shown to develop from as low as three genetic mutations [90,91]. As a proof of principle and identifying disease specific targets, Wang et al. recently developed an iPSC based de novo model of AML progression by introducing mutations in genes encoding a transcriptional regulator (*ASXL1*) followed by RNA splicing regulator (*SRSF2*) and finally a signaling molecule (*NRAS*) using clustered regularly interspaced short palindromic repeats/Cas (CRISPR/Cas) (Figure 1) [80]. The *ASXL1* represented CH, the double mutant *SRSF2-ASXL1* represented MDS and the triple mutant *SRSF2-ASXL1-NRAS* represented AML.

Hematopoietic differentiation of these iPSCs showed increasing defects in HSPCs following the addition of each mutation with failure of expression of mature myeloid markers and reduced colony-forming ability. CH and MDS cells showing decreased survival *in vitro* as compared to normal cells, while hyperproliferation was observed in AML cells. Only the AML cells possessed engraftment ability. The patient-derived iPSCs failed to recapitulate the dynamic transcriptional changes during the progression of the disease [74], which were captured by establishing the de novo disease model [80]. This study identified the transient transcriptional and chromatin changes occurring during CH and MDS. Specifically, the decreased HLA-II expression in AML as compared to MDS identified the possible mechanism of immune evasion by AML cells. The potential early AML genes identified in the study were *GATA2, MECOM, RUNX1* and inflammation related genes [80]. Identification of target genes during early phases is important for relapse and therapy resistant AML patients [92].

### 3.2. Down Syndrome-Myeloid Leukemia: An Example of iPSC-Based Sequential Disease Modeling

**Figure 2:**
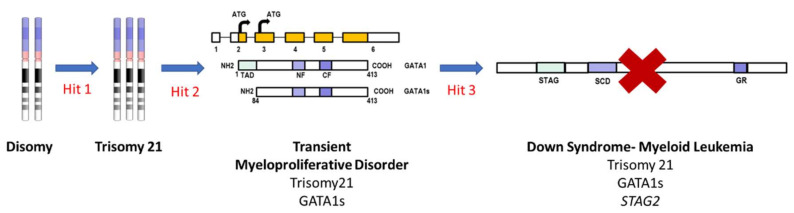
Schematic of stepwise evolution of DS-ML. The first step is trisomy 21 which augments the early hematopoiesis. In the next step, the mutation in *GATA1* results in a truncated protein GATA1s causing TMD. Acquisition of addition mutations in cohesin complex component *STAG2* resulting in loss of protein, leads to progression of TMD to DS-ML. Other cooperating mutations in genes belonging to epigenetic modifiers and signaling molecules can also lead to progression of TMD to DS-ML. TAD: trans-activation domain, NF: N-terminal zinc finger domain, CF: C-terminal zinc finger domain, STAG2: stromal antigen 2, SCD: stromalin conservative domain, GR: glutamine rich.

The genomic sequencing of DS-ML establishes that it is a unique disease in terms of sequential appearance of genetic changes as shown in Figure 2. The process of leukemogenesis in DS-ML begins with the presence of trisomy 21 that leads to aberrant hematopoiesis caused by dosage-sensitive genes on chromosome 21 implicated in leukemia predisposition such as *ERG, ETS2, RUNX1, DYRK1A, RCAN1, CHAF1B, IFNAR1, IFAR2, IFNGR2, IL10RB* and *miR-125b-2*. These genes have known functions in early hematopoiesis and increased dosage in DS children promotes upregulation of fetal hematopoietic progenitors including the megakaryocytic progenitors [88,93]. In the next step, the mutation of the X-linked gene *GATA1*, encoding a blood-specific transcription factor essential for development of the erythroid and megakaryocytic lineages caused utilization of an alternate initiation site in exon 3. This alteration produces a truncated but a functional protein, GATA1s. The truncated protein fails to downregulate proliferation-promoting genes belonging to c-MYC, JAK-STAT and MAPK-PI3K pathways that are normally repressed by GATA1. Trisomy 21 and subsequent *GATA1* mutation appears to be sufficient for TMD. The N-terminally truncated *GATA1* mutation is unique to TMD/DS-ML and is absent in DS-ALL, DS-Myelodysplastic syndrome (DS-MDS), DS infants without hematological disorders, non-DS-AML [94]. It has also been reported that the *GATA1* mutation is non-leukemogenic in the absence of trisomy 21 [95]. TMD can progress into DS-ML due to mutations observed in genes belonging to three major categories: cohesin complex components, signaling molecules and epigenetic modifiers [96,97,98].

In our lab, we have utilized iPSCs generated from fibroblast of DS children and CRISPR/Cas9 to model the stepwise acquisition of hits during DS-ML leukemogenesis. The TMD model was developed by introducing mutation in *GATA1* producing GATA1s in isogenic disomic and trisomic 21 iPSCs. The trisomy 21 and *GATA1* mutation did not interfere with the initial mesoderm differentiation. However, further hematopoietic differentiation was affected by the presence of an extra copy of trisomy 21 and subsequent mutation in *GATA1.* The trisomy 21 itself augmented the early hematopoiesis shown by increased erythroid and megakaryoid cells but was not sufficient to develop TMD. The subsequent mutation in *GATA1* led to further enhanced megakaryoid and myeloid population with significantly reduced erythroid population, mimicking the salient features of TMD [87]. Studies are in progress in our laboratory and others to generate a DS-ML model by introducing co-operating mutations in the TMD model.

## 4. Identification of Therapeutic Targets using iPSCs—Clinical and Translational Implications

The development of therapeutics for hematological malignancies largely depends on targeting the oncogenic drivers and dependencies in malignant cells following by preclinical evaluation in xenograft models. The xenograft efficacy of many drugs does not always translate to human trials. Drug development is expensive and time consuming and failure at later-stages has serious repercussions. In addition, the drugs that are effective in the clinic still have a high chance of developing drug resistance leading to disease relapse. The inclusion of tyrosine kinase inhibitors (TKIs) had great initial success in the treatment of CML harboring *BCR-ABL* chimeric oncogene, specifically in stopping the progression from chronic phase to lethal phase [99]. However, half of the initial responders experienced relapse post discontinuation of TKI treatment [100,101] since the TKI inhibitors did not target LSCs. Due to paucity and heterogeneity of samples, using patient cells to identify targets of CML-LSCs is not easy. iPSCs generated from CML cells have been utilized to generate more homogenous CML cells upon hematopoietic differentiation. The knockdown of CD156 sensitized the TKI-resistant cells to TKI treatment [102], indicating that CD156 is a unique target on TKI-resistant cells.

As described in previous sections, iPSCs derived from patient samples or harboring disease-specific mutation have enhanced our knowledge of the molecular mechanisms of disease progression and identification of specific targets. Hematopoietic differentiation of iPSCs derived from JMML and chronic myelomonocytic leukemia (CMML) demonstrated the enhanced proliferation of myeloid cells and aberrant activation of JAK/STAT or Ras/MAPK pathways [67,68,98]. MEK and Ras inhibitors suppressed proliferative capacity of HSPCs generated from CMML-iPSCs. The study also identified the liposomal clodronate as a potential treatment for CMML [103]. Similarly, the MEK inhibitor curbed the growth of JMML-iPSCs harboring the *PTPN11* mutation [69]. The identification of mutation-specific signaling pathways warrant the use of specific inhibitors in the subtypes of JMML. The iPSCs generated from JMML harboring *PTPN11* or *CBL* mutation have differential responses to the MEK inhibitors and JAK inhibitors owing to the activation of specific signaling pathways [70]. Patient cell iPSCs along with the genetic engineering provide a tool to connect specific genetic aberrations to drug responses. The MDS-derived iPSCs harboring a mutation in the gene encoding for splicing factor *SRSF2* and/or deletion of chromosome 7q (del(7q)) showed variable cellular phenotype and drug response. While the *SRSF2* mutant responded well to splicing modulators, the del(7q) cells respond to small molecule, niflumic acid implying the advantage of using iPSC in developing precision medicine [104].

Stage-specific iPSC models allow identification of drug targets in the premalignant stages and thereby possible prevention of disease progression or relapse. Severe congenital neutropenia (CN), a pre-leukemic state, is characterized by failed maturation of neutrophilic granulocytes often harboring mutations in the *ELANE* gene that encodes for elastase. About 15% of the patients do not respond to the G-CSF treatment [105]. CRISPR/Cas9 mediated knock-out of *ELANE* in iPSCs overcame the maturation arrest implying a potential therapy for CN [83]. Introduction of additional co-operating mutations in *CSF3R* or *RUNX1* in reprogrammed patient cells harboring *ELANE* mutation identified the upregulation of BAALC and phosphorylation of MK2a as key pathological events of progression of CN to CN/AML. Targeting MK2a phosphorylation using small molecular inhibitor induced cell death in mutant cells while sparing the healthy cells, thus implying a potential prevention to progression or to avoid relapse [84]. Similarly, stepwise modeling of CH, MDS and AML, identified inflammation-related transcription factors primarily present in early stages of CH and MDS and can be targeted to kill blasts that may be responsible for relapse in AML [80]. In addition, hyperactivation of innate immune signaling pathways is observed in MDS and is carried over to the AML stage. Early intervention using small molecules inhibitors for IL-1R/TLR-IRAK-TRAF6 signaling [106,107] can halt the progression of MDS to AML.

iPSC derived T cells and natural killer (NK) cells have shown potential in immunotherapy for various hematological malignancies. Nianias and Themeli have reviewed the use of iPSCs for the generation of tumor-targeting T/NK cells as cellular therapeutics [108]. The off-the-shelf T cell sources have been gaining popularity for the anti-tumor properties of T cells. There are several reports of developing an efficient method for producing cells for immunotherapy including T cell receptor expressing cells [109,110] and macrophages [111] from iPSCs. An ongoing clinical trial (Clinical trial identifier: NCT04023071) is in phase 1/1b evaluating the dose of FT516 (iPSC derived NK cells expressing high-affinity, non-cleavable CD16 Fc receptor (hnCD16)) [112] in adults with relapsed/refractory AML and B-cell lymphoma (in combination with monoclonal antibody rituximab or obinutuzumab). The preliminary data indicated that up to six doses of FT516 cells were safe and tolerable. Genetically modified iPSC (*CD38* knockout, overexpressing IL15RF, hnCD16, BCAM-CAR) derived NK cell therapy (FT576) targeting relapsed/refractory MM *in vitro* and in xenograft mouse models has shown efficacy alongside good synergy with monoclonal antibodies daratumumab/elotuzumab/anti-CD19 [113]. The clinical trials using iPSC-based CAR-NK targeting hematological malignancies are listed in Table 3. 

## 5. Hematopoietic Differentiation Approaches—2-Dimensional (2D) vs. 3-Dimensional (3D)

HSPC production from iPSCs *in vitro* is achieved using three established approaches: embryoid body (EB) formation [114,115], feeder cell co-culture [116], and extracellular matrix (ECM) coated dishes [117] capturing either the contact communication between the cells or cell–matrix interaction. The researchers have started to appreciate the presence of both of these interactions during hematopoiesis [118,119]. 2D culture systems have contributed a lot to our understanding of basic cellular functions. However, the lack of complexity of native tissue in 2D influences the cellular behavior. The differential behavior between 2D and 3D systems is mainly due to the lack of mechanical stimuli, exposure to media components, lack of cell-to-cell communication, and altered representation of cell surface markers [120]. Such differences between 3D and 2D cultures have significant effects on cellular behavior and functions, including differentiation, morphology, migration, and drug resistance.

The 3D hydrogel system contains hydrophilic polymer chains linked together and its high water content provides viscoelastic properties that are highly tunable. They have broad uses in biomedical research from drug delivery to tissue engineering. There has been published data of using natural or synthetic hydrogels to study the behavior and fate of stem cells [121]. The 3D hydrogel studies on iPSCs are focused on iPSC self-renewal, differentiation into cardiogenic lineage [122], neural lineage [123], vascular lineage [124], osteogenic lineage [125], and injection in stroke cavity [126]. The hematopoietic differentiation of iPSCs has not been studied extensively in 3D hydrogel environment. Two groups have reported the utilization of 3D hydrogels for production of blood progenitor cells using agarose [127] and self-assembling synthetic peptide hydrogels [119]. Although inexpensive and easy to crosslink, these gels suffer from mechanical instability especially when encapsulating iPSC colonies. There are no prior reports of utilizing 3D hydrogel cultures for hematological malignancies modeling. We used synthetic poly(ethylene) glycol-based hydrogel for hematopoietic differentiation of normal iPSC vs. iPSCs harboring disease specific mutation as a model for TMD [89]. The study showed significant difference in the yield of early HSPCs generated from 3D and 2D highlighting the effect of biomechanical properties on iPSC differentiation and the need to develop a more physiological relevant culture system for hematopoietic differentiation of iPSCs.

Though the use of iPSCs has enabled us to model various hematological malignancies, there are obstacles in developing certain cell types. For example, differentiation protocols to generate fully matured enucleated erythrocytes that mimic the characteristics of red blood cells are not optimized. It will be important to authenticate iPSC-derived cells by using molecular signature profiling of the desired cell type.

## 6. Perspectives and Future Directions

The failure of drugs to initiate a response, the cytotoxicity of the chemotherapeutic drugs and the relapse rate associated with hematological malignancies has been worrying clinicians for decades. The need of the hour is to determine the genetic and cytogenetic aberrations occurring during malignant transformation of normal cells, develop strategies for early interventions, and identify more effective therapeutic targets. This can only be achieved by understanding the role of various genetic and epigenetic changes in disease initiation and progression. While patient cells provide genetic context including the spectrum of mutations observed in patients, the paucity of samples and complexity of these mutations renders them useless for understanding the initiation and progression of disease as well as for studying the role of each mutation in isolation. The utility of isogenic iPSC pairs with or without the disease-specific mutation in deciphering the role of each mutation in isolation is immense. Stepwise introduction of associated mutations by genetic engineering further enables the understanding of the co-operativity between two or more genetic events in promoting oncogenesis. In addition, for some hematological malignancies, the existence of pre-cancerous mutation which aids/promotes the accumulation of other genetic events eventually leads to full blown disease. The *GATA1* mutation in TMD and *NPM1* mutations in AML are examples of such cases. The iPSC model of precancerous stage could be useful for identifying drugs that can eradicate such cells. This is an important area with respect to cancer preventative strategies and iPSC research can immensely help with this field.

Large scale sequencing studies have led to a better appreciation of the subtypes of hematological malignancies dictated by genetic or epigenetic alterations. While patient-derived xenograft models are available for major cytogenetic subtypes, such models covering the complete spectrum of human hematological malignancies are difficult to generate, owing to sample paucity and notoriety of low engraftment rates of certain subtypes. iPSCs come to the rescue in developing models representing each subtype, and even each patient—laying the foundation for personalized medicine.

While there has been a lot of effort in developing iPSC models for leukemia, similar models are either rare or non-existent in the case of MM [65] and lymphoma, respectively. Using the same principle of stepwise acquisition of aberrant changes, a sequential model can be developed using iPSC for progression of normal cells to intermediate stage of monoclonal gammopathy of undetermined significance (MGUS) and then finally MM. The stepwise progression of lymphomas is largely unknown. However, the next generation sequencing studies have identified a plethora of genetic alterations observed in lymphomas, especially the aggressive diffuse large B cell lymphoma. These models could be very useful in aiding drug development for these malignancies.

Reprogramming technology offers certain advantages: (1) It provides an expandable source of cells for studying the mechanism as well as for drug screening. (2) It can be used to model clonal heterogeneity by deriving iPSCs from single clones and understanding the role of a spectrum of mutations. (3) It can help identify novel diagnostic and therapeutic targets for disease subtypes. We envision iPSC models to complement the existing xenograft and transgenic mouse models or as a stand-alone model in cases where the generation of other models is impossible with current technology.

## Figures and Tables

**Table 1:** Examples of murine models for hematological malignancies.

Disease	Model	Outcome	Reference
Acute myeloid leukemia (AML)	Conditional transgenic targeting *NRAS* and *BCL-2*	MDS/AML transformation	[7]
	*Npm1c/Dnmt3a* mutant knock-in mice	Epigenetic therapy in preleukemic stage	[8]
	Humanized NSG xenograft expressing BCR–ABL1 and MLL-AF9	Efficient engraftment in humanized niche	[9]
	Cell line-derived xenograft model	Epigenetic therapy in pediatric AML	[10]
Acute lymphoblastic leukemia (ALL)	Cre-recombinase-inducible mouse model for *PRDM14*	Rapid onset T-ALL model	[11]
	Humanized NSG xenograft expressing *MLL-AF9*	Efficient engraftment in humanized niche, efficacy of the I-BET151 inhibitor	[9]
	T-ALL xenograft model	Targeted monoclonal antibody against NOTCH1	[12]
	*CD81* knockout cell line xenograft	Role of CD81 in homing and engraftment	[13]
Chronic myeloid leukemia (CML)	Transposon-based insertional mutagenesis	Identification of mechanisms of blast crisis	[14]
	Conditional gene knock-out strains	Identification of tumor repressor *PTEN* in *BCR-ABL* background	[15]
Chronic lymphocytic leukemia (CLL)	NSG xenograft mice	Effect of BTK inhibitor ibrutinib	[16]
	Serial transplantation in TCL-1 transgenic mice	Efficacy of programmed cell death (PD-1) immune checkpoint inhibitors	[17]
Multiple myeloma (MM)	Vk*MYC transgenic mice	Identification of novel drugs	[18]
	*BCL2L10* transgenic mice	Recapitulation of MM phenotype for validation of new therapies	[19]
B-cell lymphoma	Conditional transgenic for *MYC* and *RAS*	Preclinical testing for CD20	[20,21]
Follicular lymphoma	Transgenic linked to Vav regulatory sequence	Development of germinal center hyperplasia followed by follicular lymphoma	[22]
Peripheral T-cell lymphoma (PTCL)	Inducible transgenic for *ITK-SYK*	Efficacy of Syk inhibitors	[23]
Cutaneous T-cell lymphoma (CTCL)	Transgenic for *IL15*	Efficacy of HDAC inhibitors	[24]

**Table 2:** Genetically engineered iPSC models for hematological malignancies.

Disease	Model	Outcome	Reference
Acute myeloid leukemia (AML)	*SRSF2-ASXL1-NRAS* triple mutant	Mechanism of clonal evolution and identification of early target genes	[80]
	*RUNXI* S291fs300X mutant	Blocked granulocytic differentiation via CEBPA downregulation	[81]
	*RUNX1-RUNX1T1* fusion	Blocked granulocytic differentiation via altering the acetylome during differentiation	[82]
Congenital neutropenia (CN)/AML	*ELANE* mutant knock-out	Revert the maturation arrest	[83]
	*CSF3R* or *RUNX1* mutant	MK2a phosphorylation targeting	[84]
Polycythemia vera (PV)	*JAK2* V617F mutant	Erythrocytosis and thrombocytosis; interferon alpha and arsenic trioxide	[85]
	*JAK2* exon 12 N542-E543del mutant	Erythrocytosis; interferon alpha and arsenic trioxide therapy	[85]
Acute lymphoblastic leukemia (ALL)—pediatric	*ETV6-RUNX1*	Initiation model during fetal development	[86]
Transient myeloproliferative disorder/Down syndrome myeloid leukemia—pediatric	Trisomy 21 + *GATA1* mutant	Initiation and progression model	[87,88,89]

**Table 3:** Clinical trials of iPSC derived CAR-NK cells in hematological malignancies.

Therapy	Features	Disease	Clinical Trial Identifier
FT516	NK cells expressing hnCD16	AML	NCT04023071
	NK cells expressing hnCD16 + mAB (rituximab or obinutuzumab)	B-lymphoma	NCT04023071
FT596	NK cells expressing hnCD16, IL15RF + mAB (rituximab)	NHL, DLBCL, HGBCL	NCT04555811
	NK cells expressing hnCD16, IL15RF +/− mAB (rituximab or obinutuzumab)	CLL, B-lymphoma	NCT04245722
iCAR NK Cells	Anti-CD19	B-lymphoma	NCT03824951
FT819	A novel 1XX CAR targeting CD19 inserted into the T-cell receptor alpha constant (TRAC) locus and edited for elimination of T-cell receptor (TCR) expression	CLL, B-lymphoma, B-ALL	NCT04629729

## Data Availability

Not applicable.

## Abbreviations

ALL	Acute Lymphocytic Leukemia
AML	Acute Myeloid Leukemia
B-ALL	B-cell Acute Lymphocytic Leukemia
cALL	Children Acute Lymphocytic Leukemia
CAR	Chimeric Antigen Receptor
CH	Clonal Hematopoiesis
CLL	Chronic Lymphocytic Leukemia
CML	Chronic Myelogenous Leukemia
CMML	Chronic Myelomonocytic leukemia
CN	Congenital Neutropenia
CTCL	Cutaneous T-cell Lymphoma
DLBCL	Diffuse Large B Cell Lymphoma
DS-ML	Down Syndrome-Myeloid Leukemia
ET	Essential Thrombocythemia
FPD/AML	Familial Platelet Disorder/Acute Myeloid Leukemia
GM-CSF	Granulocyte-Macrophage Colony-Stimulating Factor
HGBCL	High-grade B-cell Lymphoma
hnCD16	High-affinity, Non-cleavable CD16 Fc receptor
HSC	Hematopoietic Stem Cells
HSPC	Hematopoietic Stem and Progenitor Cells
IL15RF	Interleukin 15 Receptor Fusion
iPSC	Induced Pluripotent Stem Cells
JMML	Juvenile Myelomonocytic leukemia
LSC	Leukemic Stem Cells
mAB	Monoclonal Antibody
MDS	Myelodysplastic Syndrome
MGUS	Monoclonal Gammopathy of Undetermined Significance
MM	Multiple Myeloma
MPN	Myeloproliferative Neoplasm
NHL	Non-Hodgkin Lymphoma
NK	Natural Killer Cells
NS/JMML	Noonan Syndrome/Juvenile Myelomonocytic Leukemia
NSG	NOD *SCID IL2Rg^null^*
PTCL	Peripheral T-cell Lymphoma
PV	Polycythemia Vera
TKI	Tyrosine Kinase Inhibitors
TMD	Transient Myeloproliferative Disorder
